# The MD Blues: Under-Recognized Depression and Anxiety in Medical Trainees

**DOI:** 10.1371/journal.pone.0156554

**Published:** 2016-06-10

**Authors:** Omar Y. Mousa, Mandip S. Dhamoon, Sarah Lander, Amit S. Dhamoon

**Affiliations:** 1 Department of Medicine, State University of New York—Upstate Medical University, Syracuse, New York, United States of America; 2 Department of Neurology, Icahn School of Medicine at Mount Sinai, New York, New York, United States of America; University of Geneva, SWITZERLAND

## Abstract

**Background:**

Mental health disease is under recognized in medical professionals.

**Objective:**

To screen medical students (MS), residents and fellows for major depressive disorder (MDD) and generalized anxiety disorder (GAD) under the new era of work hour reform with age-matched controls from a large representative cross-sectional survey.

**Methods:**

We conducted an anonymous online survey at a medical university in 2013–2014. We incorporated the Patient Health Questionnaire 2 (PHQ-2) to screen for MDD and the generalized anxiety disorder scale (GAD-7) to screen for GAD, along with additional questions on life stressors and academic performance. We compared these results to age-matched controls from the National Health and Nutrition Examination Survey (NHANES) database.

**Results:**

126 residents/fellows and 336 medical students participated voluntarily. 15.1% and 15.9% of postgraduates as well as 16.4% and 20.3% of MS screened positive for MDD and GAD, respectively. When compared to national estimates, the prevalence of a positive screen for MDD was over five-fold higher in medical trainees compared to age-matched controls (16% vs. 2.8%, p<0.0001). Similarly, the prevalence of a positive screen for GAD was over eight-fold higher in medical trainees (19% vs. 2.3%, p<0.0001).The prevalence was consistently higher within age strata. 33.3% of postgraduates and 32% of MS believe there is a significant impact of depression or anxiety on their academic performance. For stress relief, one fifth of residents/fellows as well as MS reported alcohol use.

**Conclusions:**

The stresses of medical education and practice may predispose trainees to psychopathological consequences that can affect their academic performance and patient care. The current study showed a significantly higher rate of MDD and GAD positive screens in medical trainees than the prevalence in an age-matched U.S. population, despite significant work hour reform for medical trainees. Increased awareness and support services are required at all levels of medical training. We propose that the ACGME and the Institute of Medicine may consider these findings when implementing future changes to work hour regulations.

## Introduction

The World Health Organization has ranked depression as the fourth leading cause of disability worldwide [1]. In the U.S, Major Depressive Disorder (MDD) affects 14.8 million (6.7%) adults every year. In addition, 6.8 million Americans (3.1%) are affected by general anxiety disorder (GAD) [2]. The financial and societal consequences of MDD [3]and GAD include absenteeism, reduced life satisfaction, and reduced health related quality of life. The medical profession is charged with diagnosing and treating these disorders in our patients, however the mental health of medical providers has not been well studied [4].

Psychological distress among physicians was found to originate in the early years of medical school and persist throughout their careers [5]. The mental health of physicians is often discussed in terms of job satisfaction, well-being, burnout, depression, or anxiety. Depression, anxiety and mental distress affect can directly impact patient care [6]. Higher rates of depression have been found among residents who self report medical errors [7]. Medical trainees have higher rates of suicidal ideation and suicide attempts compared to the general population [8]. Alarmingly, on average, one physician commits suicide every day in the US [9].

The Accreditation Council for Graduate Medical Education (ACGME) oversees the training of all medical and surgical residents and fellows in the United States. In the last decade, there has been increasing oversight and enforcement of work hour regulations, standards for attending supervision, and management of fatigue. In 2011 the ACGME enacted work hour restrictions [10] in order to mitigate fatigue, enhance quality of life, and increase patient safety. In addition, in November 2015, the ACGME sponsored a two day symposium of “Physician Well-Being” to address the health of medical trainees, faculty, and practitioners.

In our study, we focus on screening of depression and anxiety in medical trainees compared to the general population. We postulate that anxiety and depression are under recognized in medical trainees and we aim to evaluate the rates of these disorders in an era of increasing administrative oversight. Our research screened for generalized anxiety disorder and major depressive disorder among medical students, residents, and fellows in one academic institution and compared these results to screening tests from a large cross-sectional study in the United States. Secondary goals included identification of potential risk factors and coping strategies.

## Methods

### Study population

We surveyed all medical students in the College of Medicine (years 1–4; 643) and medical residents/fellows (post-graduate years1-7 from all medical specialties; 516) at a University-based program in New York. Invitations to participate in this study were distributed between October-November 2013 for MSs and September-October 2014for the residents/fellows.

### Materials and Data Collection

This project was deemed exempt from review by the State University of New York–Upstate institutional review board on October 17,2013according to federal regulations. An anonymous, voluntary online survey was electronically mailed through the graduate medical education office. See appendix A for a copy of the survey. The hypothesis of the study was not released to the participants. Trainees were not asked to report their past medical history or their medications.

### Survey Measures

The patient Health Questionnaire-2 (PHQ-2; score 0–6) is a validated screening tool for MDD (optimal cutoff score for a positive screen is ≥3, specificity 92%—sensitivity 83%). [11]. The generalized anxiety disorder scale (GAD-7; score 0–21) was used to screen for anxiety (positive screen for moderate to severe anxiety is a cutoff score ≥10, sensitivity 89%- specificity 82%) [12]. We incorporated these two scales in our survey “S1 Table”, and included questions on demographics, academic performance, and coping strategies.

### Comparison to National Estimates

The National Health and Nutrition Examination Survey (NHANES) is a large, nationally representative cross-sectional survey conducted in 2-year cycles in the U.S., focusing on health conditions and behaviors, diet, physical examination findings, and laboratory results. The sampling methodology seeks to create nationally representative estimates for the non-military, non-institutionalized U.S. population. Evaluations included interviews and mental health questionnaires. The design and methods of NHANES have been described elsewhere [11]. Written informed consent was obtained from all participants by the NHANES study, and the study was reviewed by the CDC/National Center for Health Statistics. For the comparison to national depression estimates, data were obtained from the survey cycle that was contemporaneous to the survey measure performed among medical students and residents (2013–2014), and data were limited to the same age range (18–45). The respondents’ actual or imputed date of birth was used to calculate age. The PHQ-2 was used to screen for depression. For the comparison to national anxiety estimates, data were obtained from all survey years in which anxiety was assessed (1999–2004). The anxiety measure, derived from the World Health Organization Composite International Diagnostic Interview, Version 2.1, was assessed only among ages 20–39 years.

### Statistical Analysis

The distributions of scores on the PHQ-2 and GAD-7 screening tools were represented in bar graphs. Within the study population, a positive screen for depression was defined as a PHQ-2 score of ≥3, and a positive screen for anxiety was defined as a GAD-7 score of ≥10. Distributions of characteristics of the survey population were summarized, including demographics and year of training. Then, the prevalence of a positive screen of depression was compared by levels of age, sex, and ethnicity, using a Fisher’s exact test for significance. The prevalence of anxiety was similarly compared. Then, the prevalence of a positive screen for depression was compared between the study population of medical trainees and the nationally-representative NHANES cohort, using a chi-square test for significance. Comparisons were performed among the entire age range of the study population, as well as within age strata (ages 18–24, 25–30, 31–35, and 36–45 years). A similar comparison was performed for a positive screen for anxiety between the study population and the NHANES cohort. All two-tailed *P* values of less than 0.05 were considered significant. Data analysis was performed using the Statistical Package for the Social Sciences (SPSS) v19 and SAS version 9.4.

## Results

**Fig 1:**
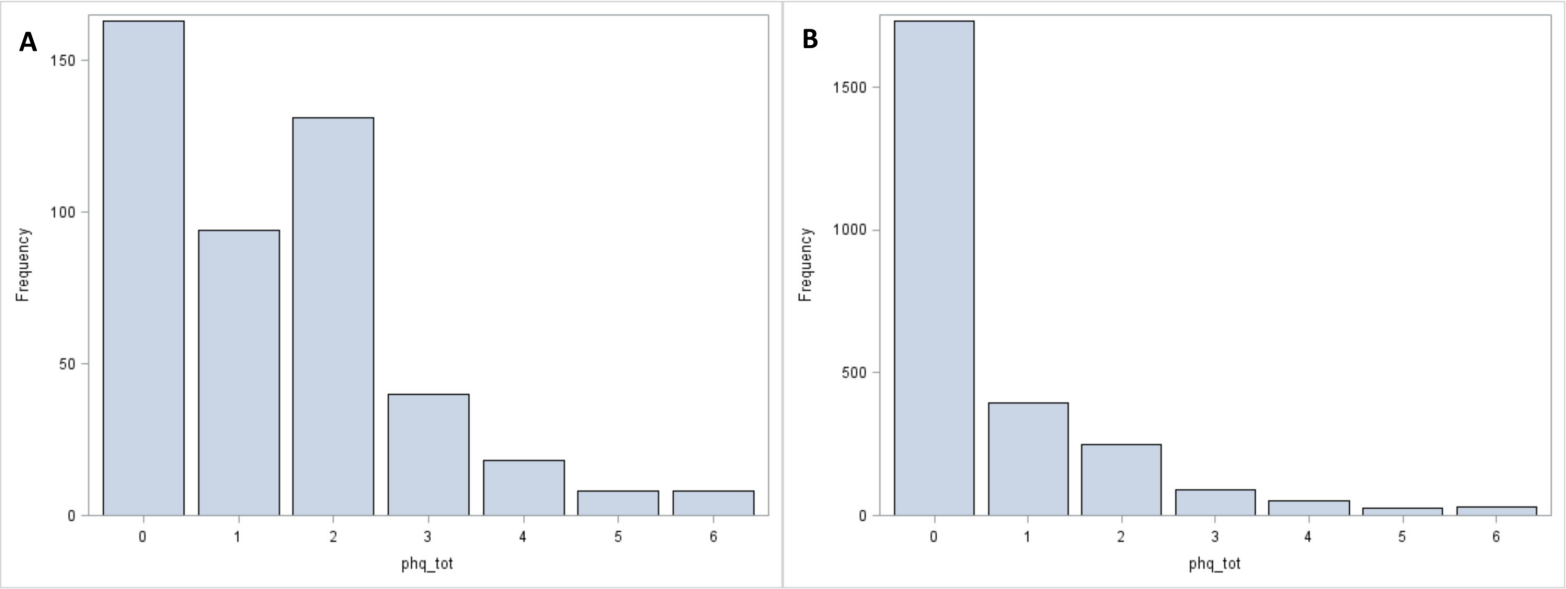
A. Distribution of PHQ-2 scores in study population. B. Distribution of PHQ-2 scores in NHANES population (ages 18–45 years).

**Fig 2:**
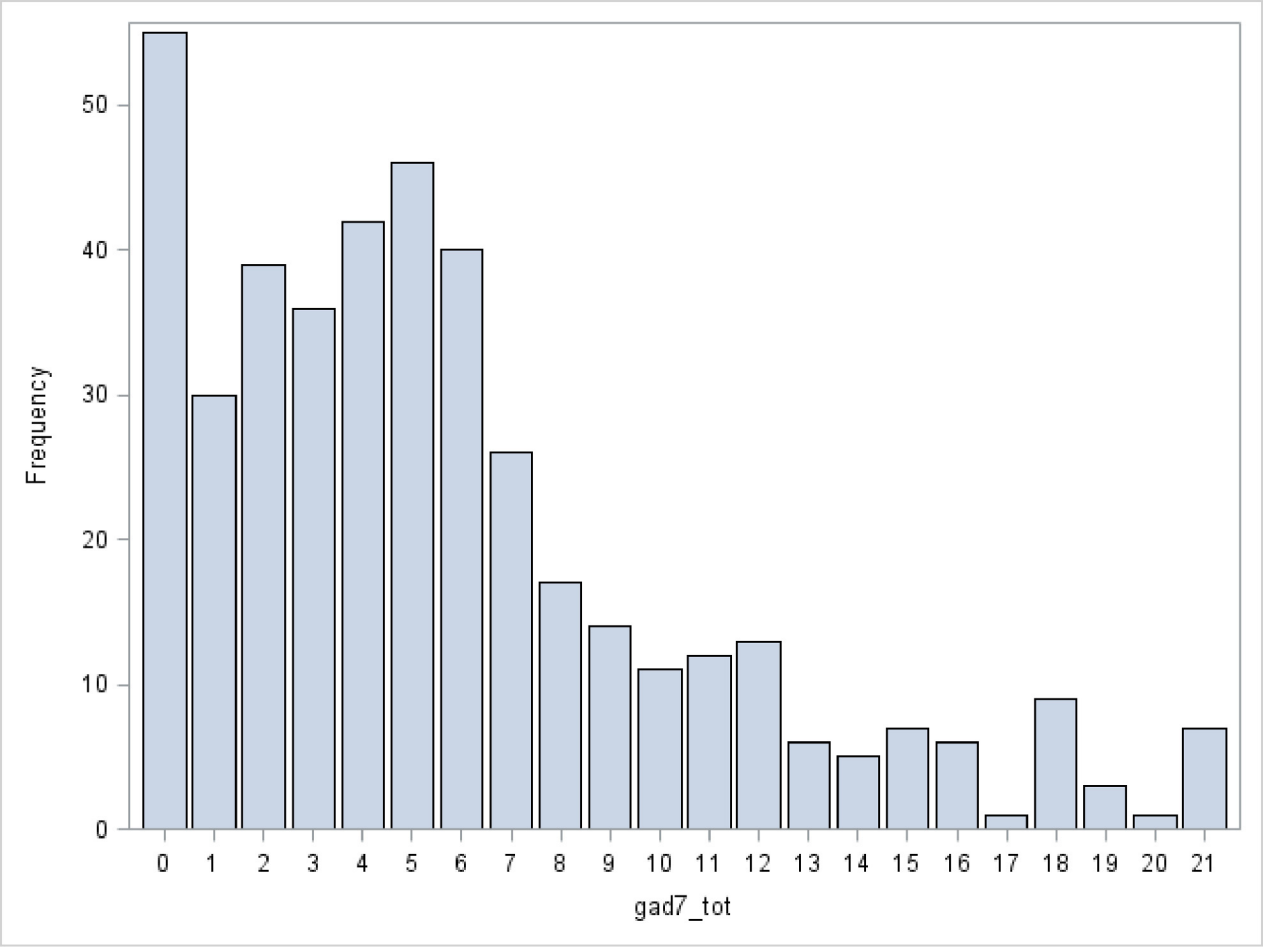
Distribution of GAD-7 scores in study population.

Three hundred and thirty six (52.3%) of MS and 126(24.4%) of medical residents/fellows completed the survey. Characteristics of the combined cohort are shown in “Table 1”. There were no significant differences in the prevalence of positive screens for depression or anxiety within levels of age, sex, or race/ethnicity (results not shown). In the study population, distributions of scores are shown in “Fig 1A (PHQ-2) and Fig 2 (GAD-7)”, and the distribution of PHQ-2 scores in NHANES is shown in “Fig 2B”. Thirty-six students did not complete the GAD-7 part of the survey.

**Table 1:** Characteristics of the study population.

Variable		Frequency, No. (%)
Sex:		
	Male	229 (49.6)
	Female	233 (50.4)
Age:		
	18–24	158 (34.2)
	25–30	233 (50.4)
	31–35	53 (11.5)
	36–45	18 (3.9)
Race/ethnicity:		
	White	296 (64.1)
	Black	30 (6.5)
	Asian	95 (20.6)
	NHPI*	13 (2.8)
	Other	28 (6.1)
Year of training:		
	MSI	85 (25.3)
	MS II	78 (23.2)
	MS III	81 (24.1)
	MS IV	92 (27.4)
	PGY-1	40 (31.8)
	PGY-2	30 (23.8)
	PGY-3	18 (14.3)
	PGY-4	16 (12.7)
	PGY-5	14 (11.1)
	PGY-6+	8 (6.4)

*NHPI, Native Hawaiian or Pacific Islander; PGY, post-graduate year

Among MS, 16.4% (55/336) and 20.3% (61/300) had a positive screen for depression and GAD, respectively. Thirty-two percent (96/300) of MS believed there is a significant impact of depression or anxiety on their academic performance. While the majority of MS deal with stress by physical exercise or talking to friends, 20% (60/300) reported that they use alcohol for stress relief and 4% (12/300) use tobacco “Table 2”. As a response to an open-ended question, 11% (33/300) talked to a counselor, 5.3% (16/300) sought psychiatric help and 9% (27/300) used prescription medications. Attending physicians and residents were the healthcare personnel who contributed to the most stressful experiences for medical students.

**Table 2:** Dealing with stress, anxiety, emotions, depression.

Actions*	Medical Students Years 1 to 4 N = 300	Residents/Fellows PGY-1 to 7 N = 126
**Talking to friends**	235	89
**Exercise/ Sports**	204	68
**Alcohol**	60	27
**Talk to a counselor**	33	7
**Prescription Medications**	27	3
**Religion**	17	4
**Psychiatrist**	16	5
**Smoking**	12	4
**Family/Spouse**	12	7
**Music**	10	2
**Movies/TV**	9	5
**Eating**	3	0
**Sleep**	2	7
**Video Games**	2	0
**Baking**	1	0
**Yoga**	0	2
**Reading**	0	2
**Vacation**	0	2
**Marijuana**	0	1
**Repression**	0	1

*Actions, based on responses to an open ended question.

Residents in the first 3 years of training participated more frequently in the survey (69.8%) than the fellows. Among residents/fellows, 15.1% (19/126) and 15.9% (20/126) had a positive screen for depression and GAD, respectively. One third (42/126) believed there was a significant impact of depression or anxiety on their academic performance. One in five residents/fellows (27/126) reported the use of alcohol for stress relief and one resident admitted to the use of marijuana. Only 4% (5/126) reported that they referred to a psychiatrist when needed. They also reported that attending physicians (54%) and the nursing staff (38.9%) contributed to the most stressful experiences during their training “Table 2”.

When compared to national estimates, the prevalence of a positive screen for MDD was over five-fold higher in the study population (residents/fellows and MS) compared to an age-matched nationally representative cohort (16% vs. 2.8%, p<0.0001), and prevalence was consistently higher when examined within age strata “Table 3”. Similarly, the prevalence of a positive screen for GAD was over eight-fold higher in the study population compared to the NHANES population (19% vs. 2.3%, p<0.0001), and the prevalence was consistently higher within age strata “Table 4”.

**Table 3:** Comparison of positive depression screens between study population (years 2013–2014) and NHANES population (years 2013–2014).

		Positive depression screen (^#^PHQ-2 ≥ 3)		
		No. (%)		
		Study Population (*N* = 462)	NHANES (*N* = 7011)	p-value
Ages	18–45 years	74 (16.0)	198 (2.8)	< 0.0001
Age strata (yr):				
	18–24	29 (18.4)	55 (6.4)	< 0.0001
	25–30	34 (14.6)	29 (5.16)	< 0.0001
	31–35	9 (17.0)	32 (6.6)	0.007
	36–45	2 (11.1)	82 (7.9)	0.6

^#^PHQ, Patient health questionnaire.

**Table 4:** Comparison of positive anxiety screens between study population (years 2013–2014) and NHANES population (years 1999–2004).

		Positive anxiety screen		
		No. (%)		
		Study Population (GAD-7 ≥ 10) (*N* = 426)	NHANES (^#^CIQGAD) (*N* = 2221)	p-value
Ages	20–39 years	81 (19.0)	50 (2.3)	<0.0001
Age strata (yr);				
	20–24	32 (21.8)	8 (1.34)	<0.0001
	25–30	40 (18.8)	13 (1.9)	<0.0001
	31–35	9 (18.4)	11 (2.1)	<0.0001
	36–39	0/16	18 (4.4)	1.0

^#^CIQGAD, Composite Interview Questionnaire Generalized Anxiety Disorder.

## Discussion

We hypothesized that MDD and GAD are under recognized in medical trainees. Furthermore, the social stigma of depression and anxiety may complicate the diagnosis and treatment of these disorders in healthcare professionals [13]. Our results suggest that medical students, residents, and fellows have five to eight times higher rates of positive screens for depression and anxiety than the rates of these diseases in an age-matched U.S. population. We highlight the need for mental health resources for medical trainees so that our future physicians can lead productive, successful lives.

Over the last two decades there has been increasing oversight of medical training in the United States. The ACGME imposed duty hour limitations for house officers in 2003[14] and again in 2011.Similarly, medical schools have implemented several initiatives, such as Wellness Programs in order to address the emotional and mental health of students. Our study shows that medical students, residents and fellows continue to have high rates of positive screens for depression and anxiety in the U.S. despite these interventions.

In a sub-analysis, there was no significant difference in age groups, gender, ethnicity or PGY level in terms of PHQ-2 or GAD-7 scores. Very few studies compared rates of depression and anxiety across different levels of medical training in the same institution. We show that preclinical medical students have higher rates of positive screens for depression and anxiety than students in the clinical years. In addition, third year medical students continue to have high rates of positive screens for anxiety. This finding highlights the early existence of these disorders in medical training, and extra attention may need to be given at this level. Another study showed the persistence of depression scores over time during medical school, suggesting that emotional distress is chronic and persistent rather than episodic [15].

As for house officers, surprisingly the residents in the first year of their training were found to have lower rates of positive screens for depression and anxiety when compared to second and third year residents. This is possibly related to increased oversight by senior residents and attending physicians with the ACGME work hour regulations. However, advanced postgraduates had higher rates of positive depression screens.

### Depression and Anxiety assessment tools

Many tools that assess for depression and anxiety symptoms or disorders have been used over the past 40 years. Previous studies used screening tools for depression that have lower specificity [16]. While other studies reported the use of screening tools with lower diagnostic threshold as the reason behind having high rates of depression and anxiety among trainees and medical students in the US, our study showed that the rates remain high even when we used a more reliable screening tool with high specificity (PHQ-2 and GAD-7). The NHANES database also used reliable diagnostic tools for MDD and GAD, as described previously.

### Dealing with Stress

Acknowledgment and treatment of these serious illnesses in medical trainees is crucial as they ultimately impact quality of life, medical education, and patient care. Previous studies showed that seeking help either through a psychiatrist or a psychologist is minimal, and 60% of physicians with recent history of suicidal ideation were unwilling to seek help due to future career concerns [17]. Our results revealed that the rates of seeking help continue to be low, with high rates of alcohol use as a method to relief stress. These results suggest that stigmatization of mental health disorders continues to persist among healthcare providers.

### Influence on academic performance

Medical students with MDD have been reported to have significantly lower grade averages [18]. In addition, there are higher rates of perceived medical errors, actual medication errors and stress during training among those who screened positive for depression. Those residents were more likely to be dissatisfied with their medical career [19]. In our study, one third of respondents felt that there was a significant impact of depression or anxiety on their academic performance. Perhaps a multifaceted approach should be used in counseling trainees who are having academic difficulties, and exploring the psychological impact on their performance.

### Intervention

For medical trainees, stigma, mental health literacy, concerns about privacy, accessibility of services, and lack of time are barriers to seeking help [20,21]. Future studies should focus on cost-effective multidisciplinary interventions to promote trainee wellness that involves screening, increasing access to services, and support during the training period of medical professionals. Similarly, support services should be considered for medical professionals in practice.

## Limitations

We believe that our results from a single institution may be generalizable to medical trainees in University-based programs across the United States. Nevertheless, multi-institutional data may be considered in order to more completely elucidate institutional, regional, and cultural differences involved in medical training. In addition, it is important to highlight that the screening tools we used in our survey cannot confirm a diagnosis of MDD or GAD. Nevertheless, PHQ-2 and GAD-7 scales have sufficient sensitivity and specificity to be used broadly in outpatient practices across the United States. In addition, data was obtained by a voluntary survey and we were not able to obtain a 100% response rate. As with all surveys there may be response bias that can confound our data.

## Conclusion

Physicians often look at the medical school and residency training as the most emotionally draining period of their careers [22]. In an era of work hour reform, our positive screens for MDD (five times) and GAD (eight times) during medical school and residency were significantly higher than the prevalence of these diseases in the general U.S. population. Raising awareness among medical students and trainees of the high prevalence of psychological distress and mental health issues may de-stigmatize these diagnoses and facilitate open discussion of the psychological effects of their training on their life. This may also facilitate diagnosis and treatment of depression and anxiety in our trainees. Also, an open discussion of the rigors of medical training may allow for more adaptive coping strategies during medical school and residency. We recommend screening of medical students and postgraduates periodically during training, which may be beneficial to the individual, the healthcare organization, and patients. Further studies are needed to identify personal and institutional related aspects that contribute to depression and anxiety and further explore the relationship between these stressors and academic performance.

## Supporting Information

S1 Table Survey. (DOCX)

## Abbreviations

MS: Medical students
MDD: Major depressive disorder
GAD: Generalized anxiety disorder
PHQ-2: Patient Health Questionnaire 2
ACGME: Accreditation Council for Graduate Medical Education
LCME: The Liaison Committee on Medical Education
