# Supplementary material for: The MD Blues: Under-Recognized Depression and Anxiety in Medical Trainees
Source: PLoS One. 2016 Jun 10;11(6):e0156554. doi: 10.1371/journal.pone.0156554 (PMC4902257; doi:10.1371/journal.pone.0156554)
Supplement: S1 Table — (DOCX) [file pone.0156554.s001.docx]

**Appendix A:**

**- Survey–**

**1. What is your age?**



18 to 24



25 to 30



31 to 35



36 to 40



41 to 45



> 45

**2. What is your gender?**



Female



Male

**3. What is your ethnicity?**



White



Black or African-American



Asian



Native Hawaiian or other Pacific Islander



Other

**4. Over the last 2 weeks, how often do you havelittle interest or pleasure in doing things?**



Not at all



Several days



More than half the days



Nearly every day

**5. Over the last 2 weeks, how often do you feel down, depressed, or hopeless?**



Not at all



Several days



More than half the days



Nearly every day

|  |  |  |
| --- | --- | --- |

**6. Over the last 2 weeks, how often have you been bothered by any of the following problems?**

**A . Feeling nervous, anxious or on edge.**

| Several days  More than half the days  Nearly every day  Not at all |
| --- |

**B. Not being able to stop or control worrying.**

| Several days  More than half the days  Nearly every day  Not at all |
| --- |

**C. Worrying too much about different things.**

| Several days  More than half the days  Nearly every day  Not at all |
| --- |

**D. Trouble relaxing**

| Several days  More than half the days  Nearly every day  Not at all |
| --- |

**E. Feeling nervous, anxious, or on edge.**

| Several days  More than half the days  Nearly every day  Not at all |
| --- |

**F. Becoming easily annoyed or irritable.**

| Several days  More than half the days  Nearly every day  Not at all |
| --- |

**G. Feeling afraid as if something awful might happen.**

| Several days  More than half the days  Nearly every day  Not at all  **H. Do you feel that your anxiety and/ or depression is affecting your academic performance?**  Yes  No |
| --- |
|  |

**7. How do you deal with stress, anxiety, emotions, depression...etc**

| Talk to a counselor  Psychiatrist  Prescription Medications  Smoking  Alcohol  Exercise/ Sports  Talking to friends or family.  Other _____(free text)  **8. If applicable: In your opinion which rotation or part of your job has been most stressful?** |
| --- |
| (Free text box) |

**9. In your opinion, which of the following personnel lead to your most stressful experience?**



Attending physicians.



Fellows.



Residents.

Interns.



Students.



Nursing staff.



Patients.

|  |
| --- |

Survey developed using www.surveymonkey.com
